# Developing a Modular Unmanned Aerial Vehicle (UAV) Platform for Air Pollution Profiling

**DOI:** 10.3390/s18124363

**Published:** 2018-12-10

**Authors:** Qijun Gu, Drew R. Michanowicz, Chunrong Jia

**Affiliations:** 1 Department of Computer Science, Texas State University, San Marcos, TX 78666, USA; qijun@txstate.edu; 2 Department of Environmental Health, Harvard T.H. Chan School of Public Health, Boston, MA 02115, USA; michanow@hsph.harvard.edu; 3 School of Public Health, University of Memphis, Memphis, TN 38152, USA

**Keywords:** Unmanned aerial vehicle, drone, air pollution, air monitoring, modular design

## Abstract

The unmanned aerial vehicle (UAV) offers great potential for collecting air quality data with high spatial and temporal resolutions. The objective of this study is to design and develop a modular UAV-based platform capable of real-time monitoring of multiple air pollutants. The system comprises five modules: the UAV, the ground station, the sensors, the data acquisition (DA) module, and the data fusion (DF) module. The hardware was constructed with off-the-shelf consumer parts and the open source software Ardupilot was used for flight control and data fusion. The prototype UAV system was tested in representative settings. Results show that this UAV platform can fly on pre-determined pathways with adequate flight time for various data collection missions. The system simultaneously collects air quality and high precision X-Y-Z data and integrates and visualizes them in a real-time manner. While the system can accommodate multiple gas sensors, UAV operations may electronically interfere with the performance of chemical-resistant sensors. Our prototype and experiments prove the feasibility of the system and show that it features a stable and high precision spatial-temporal platform for air sample collection. Future work should be focused on gas sensor development, plug-and-play interfaces, impacts of rotor wash, and all-weather designs.

## 1. Introduction

Improving air quality requires new technologies to better identify and characterize distributed pollutant sources and assess human exposure. Air pollution has been identified as a leading risk factor for the global disease burden [1,2]. In response, air monitoring networks have been established to monitor concentrations of air pollutants in the ambient air. However, the current networks are often considered insufficient to cover large areas, account for new source regimes, or implement effective pollution control strategies. The Internet of Things (IoT), defined as the network of connected air pollution sensors, has been commercialized to gather air quality data by consumers worldwide [3,4]. The latest IoT solutions need to deploy massive sensors on city infrastructures [5]. As sensors are fixed, they are only able to capture data with low spatial resolutions and cannot easily track the change of air pollution in the spatial dimension. Dedicated vehicle-based sampling systems have improved spatial coverage and demonstrated source-characterization capabilities; however, these applications can be limited by site access, proper orientation to the source (e.g., upwind vs. downwind), complex topography, or public road networks, and require extremely sensitive and expensive instrumentation. Furthermore, vertical atmospheric measurements are critically necessary for air pollution forecasting and evaluation, particularly in mega-cities with high-rise buildings. Capturing the spatial and temporal variability of aerosol particles and gases between the surface and 300 m is currently limited by the fixed sampling platforms. This situation significantly limits the understanding and management of air pollution and increases the need for the on-site fast analysis of field samples.

The unmanned aerial vehicle (UAV) is becoming an attractive experimental platform for high-spatial-resolution, near-surface vertical profiling of atmospheric pollution in recent years [6]. Previous technologies used balloons, aircraft, and satellite remote sensing, which are unmaneuverable or expensive [7,8]. Recent advancements in consumer UAV technology present a low-cost solution for sampling the lower troposphere, taking advantage of their abilities to maneuver in both the horizontal and vertical dimensions and to hold a fixed position in the air even under high-wind conditions [9,10]. Mobile real-time low-cost commercial micro-sensors equipped with new technologies of consumer UAVs offer numerous advantages for capturing the spatial and temporal variability of air pollutants and provide the ability to measure important air pollutants with high sensitivities and temporal resolutions [11]. While the potential of UAVs for air quality monitoring is promising, adoption of them has been limited by proper UAV designs, adequate sensor technologies, and regulatory uncertainty [12].

UAVs have demonstrated their applications in a few air pollution monitoring studies [13,14,15,16,17]; however, seldom has a study adequately addressed the technological challenges frequently encountered in the real-world monitoring. Specifically, a UAV-based air monitoring platform should address these challenges: (1) Low-cost consumer UAVs. Previous applications used proprietary professional UAVs that do not allow the general users to customize their needs. These UAV platforms are expensive and require specialized design and maintenance. (2) Synchronization of monitoring sensor data and GPS data. For real-time monitoring and geospatial data modeling, air pollutant data and GPS data need to be synchronized, as they come from two separate components. However, the internal clocks of the sensor and the UAV are not synchronized at all. Worse, they do not have the built-in ability to implement network timing protocols. Rather than synchronizing the devices, we study and develop mechanisms to produce synchronized data within an error tolerance. (3) Multiple air pollutants. Air pollutants are present as mixtures in all the air environments. Both the scientific community and regulatory agencies have been shifting from the traditional single-pollutant approach toward a multi-pollutant approach to quantify the health consequences of air pollution mixtures [18]. This trend requires a platform that can house and integrate multiple sensors with different operating principles [19]. (4) Energy efficiency and flight time. A typical consumer UAV in the price range of $500 to $2000 can fly for about 15 to 30 min on one fully charged battery. Carrying additional devices may reduce the UAV flight time, as the onboard devices add weights to the UAV and the devices themselves consume electricity. A typical USB-powered micro-controller device consumes power in the range of 1 W to 10 W. It is necessary to study and find a design with hardware and software to reduce the weight and energy consumption of the UAV system. (5) Safety and restrictions in cities. Importantly, the UAV’s flight paths need safe airspace to avoid many obstacles in a city environment, such as buildings, lights, power distribution lines, trees, no-fly zones and so on. Also, UAVs cannot be deployed without restrictions. Under current aviation safety operating regulations, restrictions are placed on their use in commercial, research, and private applications.

To address these challenges, we designed and developed a consumer UAV-based air monitoring system. Rather than designing a customized UAV system, we targeted the building of a system with all off-the-shelf consumer components, including both hardware and software. Our contribution is a modular design that enables a consumer UAV platform to carry multiple sensors and be capable of real-time monitoring multiple air pollutants. This UAV system features a stable and high precision spatial-temporal platform for air sample collection. We also tested the feasibility and capability of current consumer UAVs for air quality monitoring applications in the field.

## 2. Design of the Platform and Field Tests

### 2.1. The Modular Design

A UAV system typically consists of the UAV itself, a ground station, and a few onboard gadgets, such as a first-person view camera. In many applications, a UAV is simply used as a carrying platform and does not intervene with the operations of the onboard gadgets. The controls of the UAV and the gadgets are often separated as well. For air quality monitoring, however, the UAV must function beyond a simple platform that carries multiple air pollutant sensors. It is more important for the UAV to integrate the data from all onboard sensors and tag the data with geolocation information in real time. Hence, a modular design is highly desired to build a UAV system that can be easily deployed and managed in field uses.

**Figure 1:**
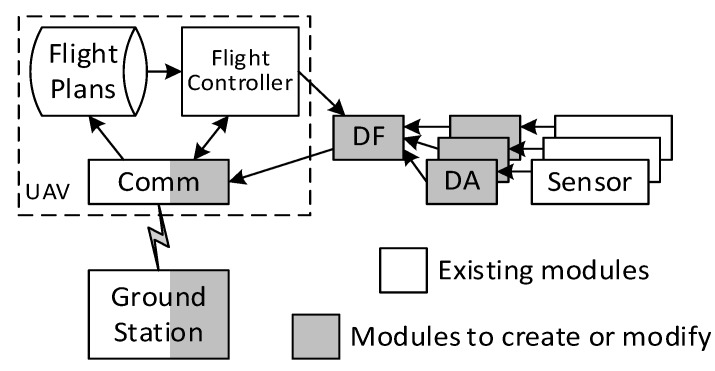
The modular design of an unmanned aerial vehicle (UAV) system with multiple air pollutant sensors.

In the modular design of Figure 1, the system comprises five modules: the UAV, the ground station, the sensors, the data acquisition (DA) module, and the data fusion (DF) module. The operations of the UAV are controlled by the ground station. The UAV has a flight controller that directs the UAV to accomplish specific tasks according to a flight plan. The UAV reports its status to the ground station through its communication module. Our work was mainly focused on developing software components that synergize the existing products, as we used off-the-shelf products to build the system. Figure 1 shows the software components that are existing (in white boxes) and that need to be developed (in gray boxes) in the system.

### 2.2. Air Pollutant Sensors

Air pollutant sensors vary greatly in their working mechanisms. Each sensor is made for one specific air pollutant, such as particulate matter or nitrogen dioxide, and produces different kinds of signals. The signals are in response to the concentrations of the pollutants and typically need to be calibrated before use. Therefore, we needed to develop an individual data acquisition module that is bound to each sensor to gather the signal and produces sensor-specific data for later processing in the data fusion module. Each data acquisition module runs its own program customized for each sensor. To save battery, the data acquisition module is built into a low cost and energy efficient micro-controller. As a prototype, our UAV carried two air pollutant sensors:

(1) The particulate matter (PM) sensor (OPC-N2, Alphasense Inc., Essex, UK) counts particles optically and converts the number concentrations to mass concentrations of PM_1_, PM_2.5_, and PM_10_. It has a built-in data acquisition module and transfers data through a USB emulated serial port to external devices. The sampling period is set at 1.5 s per sample but can be adjusted in the range of 1 s to 30 s per sample. Each sample contains 62 bytes of data. This OPC sensor has been specifically tested for UAV applications [20].

(2) The nitrogen dioxide (NO_2_) sensor (NO2-B43F, Alphasense Inc., Essex, UK) is a 4-electrode gas sensor that produces current signals proportionate to the concentration of NO_2_. The NO_2_ sensor only produces analog signals. Hence, we developed a data acquisition module that converts its analog signals to digital data. The sampling period is set at 0.25 s per sample but can be adjusted in the range of 0.01 s to 100 s per sample. Each sample contains 8 bytes of data. The data acquisition module transfers data through a USB emulated serial port as well.

They are both lightweight and compact sensors and suitable to be carried on a UAV. These sensors have been field tested and recommended by the U.S. Environmental Protection Agency (EPA)’s Office of Research and Development [21].

In the modular design, the sensors do not send data directly to the ground station. Rather, we used the data fusion module to aggregate data from the sensors. Then, we used the UAV’s communication module to send the aggregated sensor data to the ground station. Thereby, we could share one communication channel with all sensors to reduce the energy and cost of the system. Depending on the data throughput required by the applications, we had two kinds of communication modules. One was the telemetry radio communication between the UAV and the ground station, which is suitable for low rate data communication but has a long-distance coverage. For high throughput data, we deployed WiFi as the communication mechanism, although the WiFi communication distance is limited in comparison to the telemetry radio communication. Because the sensor data is transmitted to the ground station in real time, we also added new software components in the ground station to receive, store, and analyze data.

### 2.3. Data Fusion

The data fusion module is the core component of this modular design. It is responsible for integrating the UAV’s geo-location data, time data, and sensor data. The UAV’s flight controller provides the geo-location data to the data fusion module. The geo-location data often come from a GPS device to which the flight controller connects. Because the GPS data carry time information, the flight controller provides time data as well. Furthermore, the flight controller can provide the UAV’s attitude data, such as orientation, velocity, acceleration, and so on, to the data fusion module. Such information, accompanying the air pollutant data from the sensors, can show how the sensor data are collected by the UAV, and will help us understand the context of the measurements.

**Figure 2:**
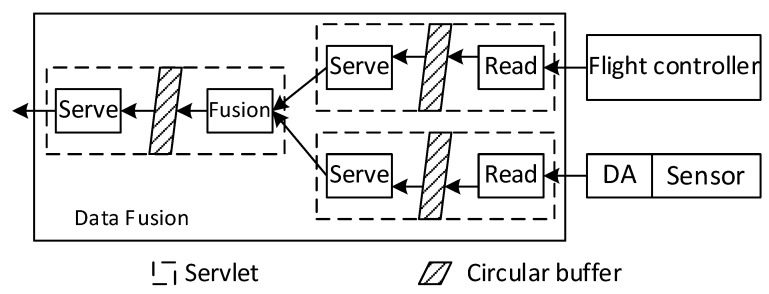
Design of the data fusion module.

Because all sensors and the flight controller provide data in different formats and at different rates, the data fusion module needed to accommodate heterogeneous data sources. Furthermore, we may need to add or remove multiple different sensors in the UAV system. To support these needs, we adopted the servlet’s programming model (A servlet is a small program that is used to extend the capabilities of servers that host applications accessed by means of a request-response programming model) for the data fusion module shown in Figure 2. The data fusion module runs two types of servlets (data source servlet and fusion servlet) to obtain data from the air pollutant sensors and the flight controller.

For each data source, a small data source servlet runs inside the data fusion module. Each data source servlet has a read thread and a serve thread. The read thread periodically reads from the data sources. One data source servlet reads the GPS data from the flight controller with a period of 0.5 s. The other data source servlets read data from the air pollutant sensors at the rates specified by the sensors. Then, the read thread stores the data and the timestamps into a circular buffer (A data structure that uses a single, fixed-size buffer as if it were connected end-to-end). Upon a request from the downstream fusion servlet, the serve thread reads the most recent data from the circular buffer pointed by the read thread. The serve thread can access the data in the circular buffer at a different rate than the read thread, which is necessary for the later fusion servlet to unify the data time from all the data sources.

A fusion servlet is placed at the downstream of the data source servlets. The fusion servlet has a similar structure to the data source servlets and includes a fusion thread and a serve thread. The serve thread provides data to the downstream applications. The fusion thread periodically reads from all data source servlets with a period of one second. When the fusion thread obtains the most recent data and the timestamps from all the data source servlets, the fusion thread checks the differences of the sensor data timestamps and the GPS data timestamp. If the time difference is smaller than a threshold, the fusion thread stores the sensor data, the GPS data, and the GPS data timestamp in a circular buffer. Otherwise, the sensor data that has a large time difference is discarded, because the sensor data was not acquired in a close time proximity of the GPS data. In our implementation, the threshold is set at 0.5 s, which is the period of GPS data. In this way, the time of stored sensor data is aligned with the time of GPS data. The alignment error is bound to 0.5 s. When the UAV flies at a maximum speed of 10 m/s, the alignment error is equivalent to a maximum location error of 5 m, and the standard deviation of the equivalent location error is 1.4 m. Such location error satisfies the spatial resolution requirement of many air quality monitoring applications.

Running these servlets decouples the fusion process from individual data sources, improves the applicability of sensors, and enables quick and easy diagnostics on failed sensors. The data fusion module is implemented in a UAV’s companion computer. With this modular design, we were able to use a variety of consumer UAVs to carry air pollutant sensors and obtain spatial-temporal data in real time.

**Figure 3:**
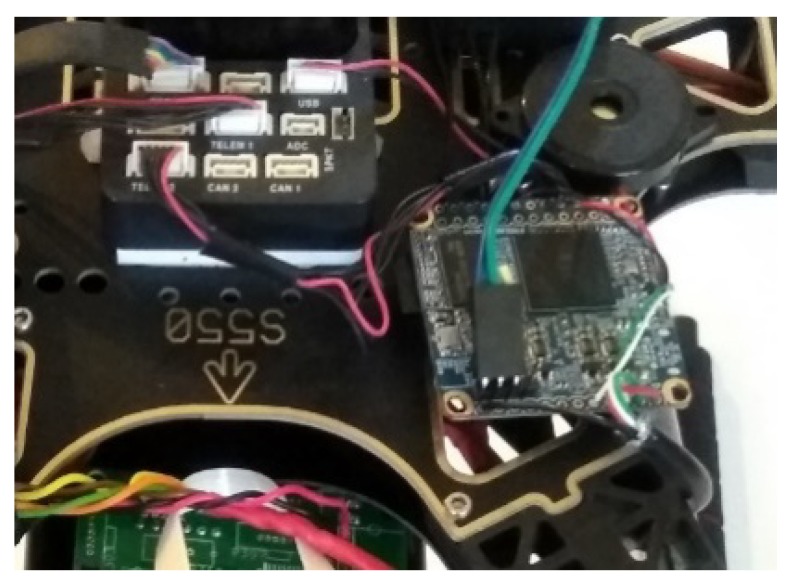
Data fusion companion computer.

We used a NanoPI Neo Air board as the onboard companion computer to implement the data fusion module (Figure 3). The NanoPI Neo Air board is a low cost embedded computer that has a powerful 1.2 GHz quad-core CPU and 512 MB memory. It has a built-in WiFi component and multiple USB ports and serial ports. We connected these ports to the data acquisition modules and the flight controller to obtain data. The NanoPI Neo Air board runs Armbian Linux and thus can import a vast pool of supporting tools and libraries. It is very suitable for developing and testing a variety of functions in data acquisition, fusion and communication modules for air monitoring applications.

Three data source servlet programs run in the onboard companion computer to collect data from the PM sensor, the NO_2_ sensor, and the flight controller. The fusion servlet program runs as well to gather data from the three data source servlets and provide the aggregated data as a service to applications. The aggregated data includes the sensor data and the UAV data (such as GPS, attitude and so on) with synchronized time stamps.

### 2.4. Data Format and Interface

Because all sensors produce different kinds of data in their formats and interfaces, unifying data specifics is another important aspect of the design. All data source servlets read raw data from the data sources in the data formats specified in their datasheets. Then, the data source servlets convert the raw data into the packed binary data format, where the values of the data (e.g., integers, floating-point numbers, and strings) are converted to bytes and then concatenated in a byte array. Such packed binary data is concise in that only the values of data are included. Meanwhile, the packet binary data is compatible with all programs, because the byte representations of the data values follow the same standards. For example, floating-point numbers are represented according to IEEE 754. The fusion servlet processes and provides data in the packed binary data format as well.

We also included a few different types of data interfaces between the modules in the system. Because many consumer UAVs have USB interfaces and run with some variants of Linux, we built a few common data interfaces into the data acquisition module and the data fusion module, such as serial RS232, USB communications device class (CDC), or human interface device class (HID), to accommodate most UAVs.

### 2.5. The Prototype UAV System

**Figure 4:**
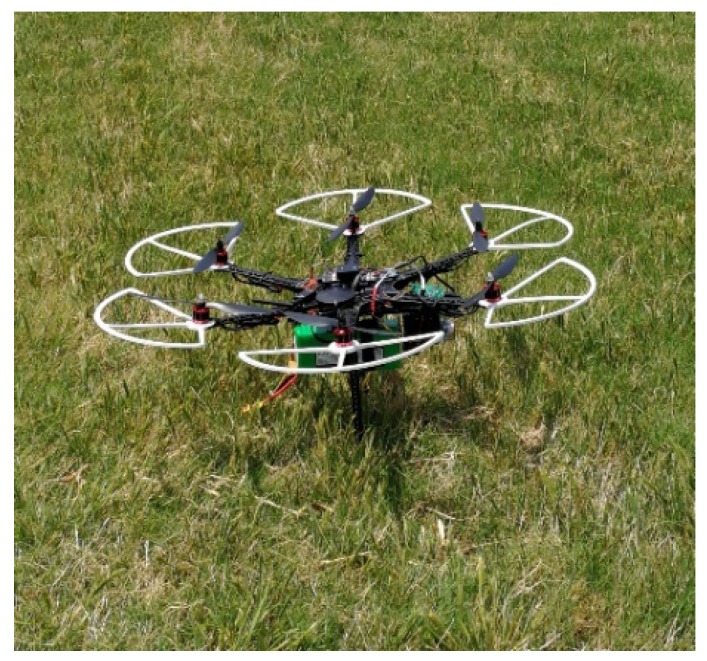
A prototype of the unmanned aerial vehicle (UAV)-based air monitoring system.

We built a prototype of the UAV system, shown in Figure 4, to test its feasibility and perform some preliminary experiments on air quality monitoring. The prototype was composed of three major components: a UAV, two air pollutant sensors, and a data fusion module. The prototype UAV was built on the S550 frame and the Pixhawk 2.1 flight controller. We also adopted open source software Ardupilot for flight and mission control.

The hardware included: (i) S550, a lightweight hexacopter frame. It is based on an upgraded design of DJI F550 hexacopter and has sufficient load space under the frame. (ii) Pixhawk 2.1, the latest flight controller from the open hardware Pixhawk project in collaboration with 3D Robotics and the Ardupilot group. It has a rich set of sensors, including a GPS, magnet sensor, barometer and so on. With these sensors, it provides geolocation and attitude data in real time through serial ports to the onboard companion computer. The maximum load of the UAV (excluding the UAV and battery) is above 1 km, which is enough to carry all the sensors. The sensors and the companion computer are very lightweight and only take 4.5% of the system’s total weight.

In terms of the software: (i) We deployed Ardupilot (version 3.4.6, GitHub, San Francisco, CA, USA) as the UAV’s autopilot software in its flight controller. Ardupilot is an open source autopilot software, designed and adopted for a variety of copters, planes, rovers, and submarines. It has APIs to transmit UAV data and control commands. (ii) The ground station used the open source QGroundControl version 3.3.2. It provides full flight control and mission planning for MAVLink enabled UAVs. (iii) We developed the data fusion module in the onboard companion computer. The module was implemented in C and Python and used the SDKs provided by Ardupilot and the sensors to control and communicate with the UAV flight controller and the sensors.

We developed two applications in Python for data collection and visualization. The data collection application obtains data from the data fusion module and then saves the data into CVS files in the onboard companion computer. Later, users can download the files from the companion computer to other computers for offline analysis. The visualization application is a web application built on the Flask web framework. It obtains data from the data fusion module and then provides real-time data on a web page. We set up a WiFi access point and ran the web application with the Apache web service in the companion computer. Thereby, users can access the web application through WiFi to browse the real-time air pollutant data and UAV data with their smartphones and computers. In our prototype, the data collection application saves all data, while the visualization application mainly shows the critical data in order for users to track the status of the drone and the sensors.

We adopted the off-the-shelf consumer UAV for the purpose of affordability and programmability of this platform. The cost of this UAV is only 1/3 or less of that of an industrial grade drone with a similar payload. The open source software allows the modular design to house multiple heterogeneous sensors together. In contrast, commercial drones often have close software that allows limited programmability.

### 2.6. Field Tests of the Performance

The prototype UAV was tested in different field settings to collect flight data and sensor data for feasibility checking. The goals of the tests were to find: (1) whether the sensors and the data fusion module consume a significant amount of battery power and (2) whether the accuracy of sensor data is influenced by operations of the UAV. The following four specific environmental settings were selected for the field tests. These settings were selected as they presumably had strong air pollution sources. The tests were performed on non-rainy days with wind speeds below 5 m/s. We set up the prototype UAV system with a 16,200 mAh battery to support a maximum flight time of around 30 min.

Test 1: Air pollution gradient in proximity to a busy highway. This highway had an Annual Average Daily Traffic (AADT) count of 55,000 (TN Department of Transportation). The UAV departed at 100 m away from the highway and flew an 800 m path perpendicular to the highway.

Test 2: Air pollution gradient in proximity to a busy highway intersection. This site was near an intersection of two busy highways (AADT = 157,000 and 137,000). The UAV departed from 10 m away from one highway and flew a 500 m path perpendicular to the highway.

Test 3: Air pollution gradient in proximity to a truck stop. This site was the largest local truck stop in this region and was near an interstate highway (AADT = 36,000). The UAV departed at 200 m away from the board of the stop and flew a 1000 m path.

Test 4: Vertical air pollution gradient over a restaurant chimney. The UAV flew upward from the ground and hovered for 10 s at 10, 15, 20, and 25 m.

## 3. Results and Discussion

### 3.1. Data Acquisition and Synchronization

**Figure 5:**
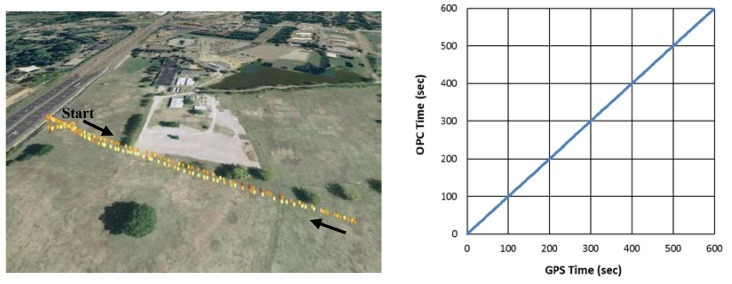
An example of data acquisition and synchronization.

The sensors and the flight controller produce data at different rates. Using the servlet programs, we were able to obtain synchronized data from the data fusion module at the rate of one sample every two seconds. Figure 5 shows the data acquisition and synchronization in Field Test 2, in which PM_2.5_ concentrations and GPS readings were collected along the flight paths. Each sample has 62 bytes, and the data throughput is very low at 248 bps. The OPC and GPS started simultaneously, and the elapsed times recorded by the GPS and OPC were the same, as indicated by an exact 45° straight line.

### 3.2. Power Consumption

**Figure 6:**
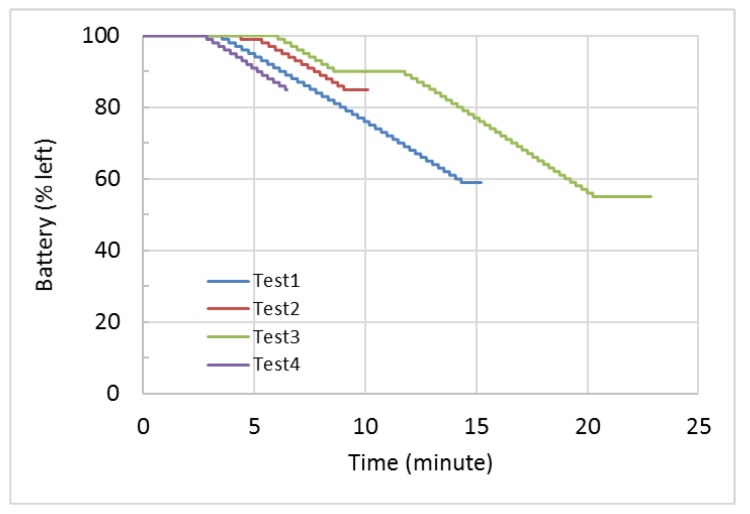
Power consumptions during idling and flight.

We measured the power consumption of the UAV system on the ground and during flight. Figure 6 shows the battery status in the four field tests. When the UAV was in the air, the consumed current reached about 30 Amp regardless its maneuvers and speed. In contrast, when the UAV was on the ground, the consumed current was only about 1 Amp. Because the battery voltage is around 16 V, the power consumption was about 480 watts during flight but only 16 watts on the ground. We also measured the power consumption of the sensors and the companion computer. They consumed about 5 watts in total, which is almost negligible compared to the UAV’s power consumption. Hence, the addition of the air pollutant sensors has insignificant effects on the flight time and operations of the UAV in our prototype. The battery displayed consistent linear consumptions in multiple tests. The consumption rate was 3.73% per min, giving a total flight time of 27 min with a full charge. We also did one endurance test to measure the actual maximum flight time, which was 32 min. The measured maximum flight time gave us an estimated battery capacity of 15,700 mAH, which was close to the marked full battery capacity of 16,200 mAH. Hence, the estimated maximum flight time was consistent with the endurance test result and the battery capacity.

### 3.3. Accuracy of Air Pollution Data

**Figure 7:**
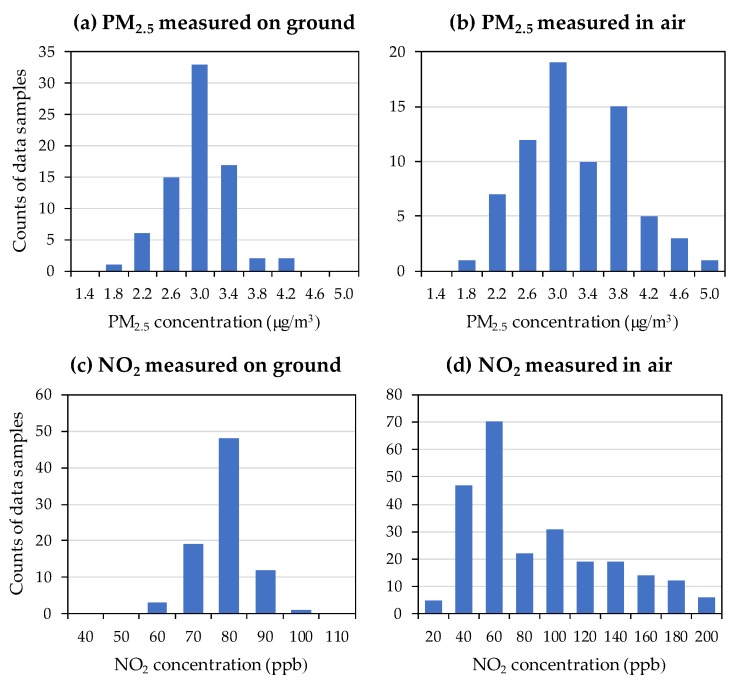
Histograms of sensor readings before and during flights.

Air pollutant sensors are designed and manufactured mostly for use in a stable environment, for example, installed onto a post or used inside a lab. Many sensors are very sensitive to changes in the environment. It is thus necessary to examine whether the sensors operate differently when being carried on a UAV. We conducted experiments to read data from the PM sensor and the NO_2_ sensor at the same location when the UAV was static and when it was hovering (Figure 7).

When the UAV was on the ground, both PM_2.5_ data and NO_2_ data showed a normal distribution in Figure 7a,c. The central points of the histograms are the means of the readings, and the widths of the histogram are the deviations in the readings. When the UAV was in the air, Figure 7b,d show that their histograms were changed during the flight. We observe that the histograms during flight are shifted to the right and are widened. Because NO_2_ data shows more changes than PM_2.5_ data, the NO_2_ sensor was more affected by the UAV.

We conducted further experiments to find the causes of such influences. We learned that the UAV consumes a larger current than the sensors and the data acquisition modules. Because we let the UAV and onboard devices share the same power source, the ripple in UAV’s current produced a significant noise into the sensors and the data acquisition modules, which resulted in the changes of sensor readings. Because the PM sensor uses an optical mechanism and has a built-in data acquisition module, it is more noise-resistant than the NO_2_ sensor.

### 3.4. Field Test Results

**Figure 8:**
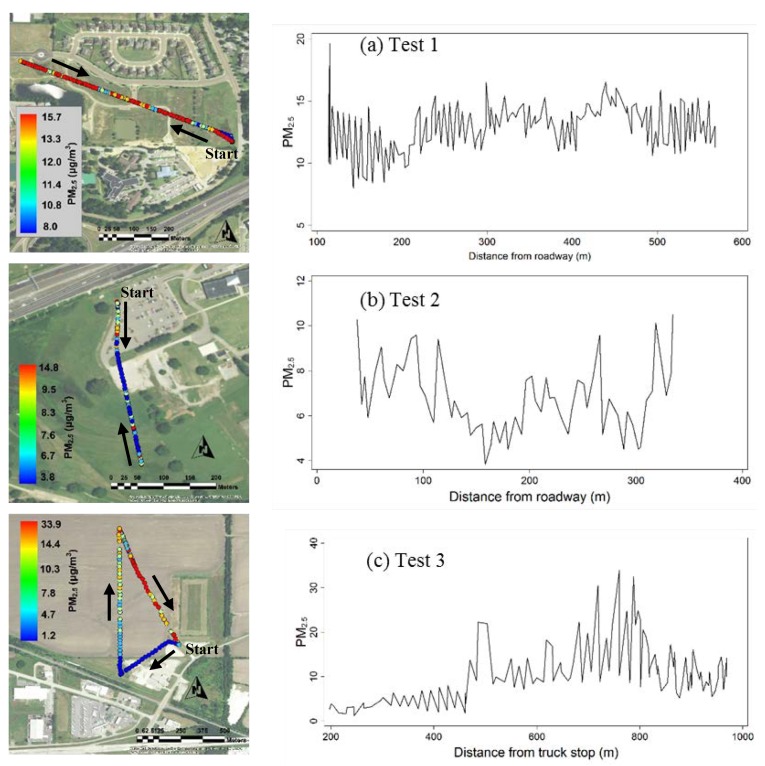
Particular matter sensor (PM_2.5_) measurements (µg/m^3^) with distance to the emission sources in Tests 1–3.

Figure 8 displays PM_2.5_ concentrations with respect to distance from the major emission sources in field tests 1–3. Only PM data were presented as the PM sensor was not affected by the UAV operation. The nearby U.S. EPA’s monitoring stations showed that the daily PM_2.5_ concentrations were 12.7, 12.0, and 12.8 µg/m^3^ on the day of test for Tests 1, 2, and 3, respectively. Our UAV measurements showed that average (±standard deviation) PM_2.5_ concentrations were 13.2 ± 2.0, 8.8 ± 3.7, and 10.5 ± 6.1 µg/m^3^ for Tests 1, 2, and 3, respectively. Our results were within the concentration ranges measured by the EPA reference method, considering the spatial and temporal variations. No clear concentration gradient was observed with distance to the source in these tests. Although PM_2.5_ concentrations presumably decrease with increasing distance away from the traffic source [22], field studies showed the increment was very small in the low PM ambient air in the US. A national review of near-road air pollution estimated that PM_2.5_ concentrations increased by 13% in near-road environments and by 15% for sites within 20 m of the roadway [23]. A Detroit study showed no difference in fine particle concentrations at 10 m and 100 m sites near a highway [24]. Given an average PM_2.5_ concentration of 12 µg/m^3^, the increment would be no more than 2 µg/m^3^ near roads, which could easily be overshadowed by sensor fluctuations during the flight.

**Figure 9:**
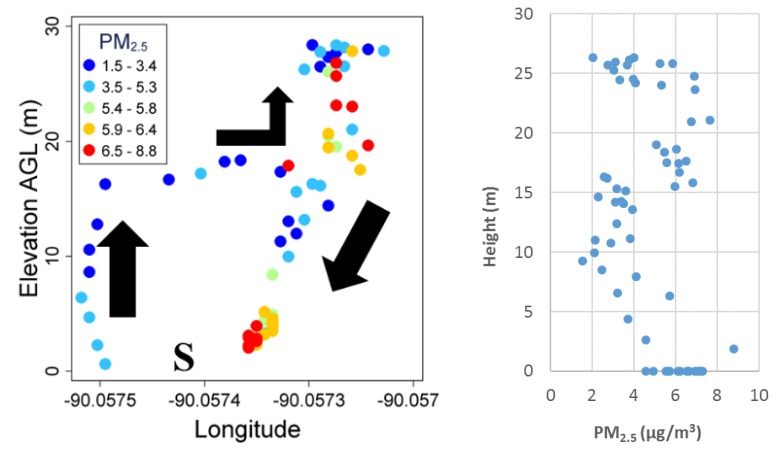
Vertical particular matter (PM_2.5_) concentration profile over a barbeque restaurant. “S” indicates the takeoff location.

Field Test 4 aimed to obtain a vertical PM profile in the ambient air near an emission source (Figure 9) where the PM_2.5_ concentrations were low, with a range between 0.5 and 9 µg/m^3^. No clear gradient was observed here either, possibly because the restaurant had no grilling activities and the stack had no smoke vented out during the test.

### 3.5. Limitations and Future Work

Our UAV-based air monitoring platform showed the ability to simultaneously collect air quality and spatial information following preset flight paths. Although this platform achieved the original design goals, a number of study limitations should be recognized. The design limitations were mainly to do with the sensors, electronic interference, and a lack of weatherproof framework. This UAV only used two air sensors, and one sensor was proved to malfunction due to interference from the UAV operation. Although more sensors could easily be achievable, additional experiments are needed to test the flying balance and power consumption. In the current design, the electronic interference from the UAV has more or less impact on the sensor readings. A better electronic design is needed to shield onboard sensors from UAV’s electronic interference. The current UAV is not waterproof, which will require a more complex design for air inlets free from water interference. Full calibration and field validation of sensors went beyond the scope of this prototype development. Sensors on dynamic UAV systems intrinsically cannot be as specified, controlled, or reproducible as more traditional air pollutant measurement methods. Although our PM sensor obtained results comparable to those from the EPA reference method, a quality control gap persists for low-cost, micro-form air pollution sensors [19]. Little is known about how the wind field impacts sensor performance, and this study did not test all the environmental factors. The coauthors’ preliminary results showed that the orientation of the inlet line to upwind matters, although previous studies indicated that rotor wash had minimal impacts on particle sampling [25]. Lastly, our limited number of field tests warrant more comprehensive investigations under different conditions.

An extensive literature review [11] only identified a small number of air pollution studies that utilized UAVs to measure air pollutants, with a focus on particulate matters, carbon dioxide, methane, and ozone. The field is still in its early stages of development, and the limitations of our prototype UAV system call for future development and improvements. As well as the two sensors tested in this study, the interest should be extended to more sensors for other criteria air pollutants (carbon monoxide, ozone, and sulfur dioxide), climate change-causing gases (carbon dioxide and methane), and oil industry-related pollutants (volatile organic compounds and hydrogen sulfide). The primary focus of future improvements would be light-weight, low-cost and reliable air quality sensors. With increasingly mature UAV technologies, a successful platform relies mainly on gas sensors suitable for UAV applications, i.e., “flying sensors” [16]. However, it is a daunting task to identify, test, and validate numerous air sensors available on the market. U.S. EPA has tested a number of emerging air quality sensors [26], but all the sensors were housed at fixed ground sites during the tests. Previous studies have examined sensor interferences from environmental factors like temperature, humidity, pressure, and other pollutants [25], but seldom examined those from drone operations as observed in this study. The use of multiple sensors also requires standard plug-and-play (PnP) interfaces like USB ports. More experiments are required to gain a full understanding of how sensor readings are impacted by wind speed, wind direction, turbulence from propellers, and orientation of air inlets. The huge amount of data generated by high-frequency sensors also suggests the need for a real-time data processing and visualization system. For example, a 3-dimensional (3D) AQI monitoring system has been developed to efficiently build real-time fine-grained 3D AQI maps [27].

## 4. Conclusions

A consumer UAV-based air quality monitoring system has been developed for measuring and displaying air pollutant concentrations in a real-time manner. The modular design enables the system to carry multiple different air pollutant sensors and integrate the data from all the onboard sensors with geolocation information in real time. In field tests, the UAV obtained concentrations of fine particulate matter comparable to those measured by the reference method, proving the capabilities of this system to operate in real-world conditions. The field tests showed that the onboard devices did not affect the UAV’s power consumption and flight time; however, the UAV operations interfered with the chemical resistant sensor during flights. Future work will include the investigation of how to shield onboard sensors from the UAV’s electronic interference, the development of area coverage flight missions, the effects of inlet positioning and rotor wash, the real-time data processing system, and the integration of UAVs with IoT systems.
